# Retraction Note: K-ras-ERK1/2 down-regulates H2A.X^Y142ph^ through WSTF to promote the progress of gastric cancer

**DOI:** 10.1186/s12885-022-10052-1

**Published:** 2022-09-07

**Authors:** Chao Dong, Jing Sun, Sha Ma, Guoying Zhang

**Affiliations:** 1 Department of Clinical Medicine, Qujing Medical College, Qujing, 655000 Yunnan China; 2 Department of Pharmacy, Qujing Medical College, Sanjiang Avenue, Economic Development Zone, Qilin District, Qujing, 655000 Yunnan China

**Retraction Note: BMC Cancer 19, 530 (2019)**

**https://doi.org/10.1186/s12885-019-5750-x**

The Editor has retracted this article. Concerns have been raised about a number of figures in this article:

- In Figure 1A and 1C it appears that the Western blot quantification doesn't match the images presented. In addition, the label for the y axis does not match the Western blot label it is supposed to quantify in 1A.
- In Figure 6 it appears that the image used for Anti-GFP in Figure 6B and 6C are the same.

The authors stated that the figures were obtained through a commercial lab hired to perform some of the experiments for this study. The authors didn't receive the raw data and couldn't share it with the journal. They were also unable to reproduce the findings in their own follow up experiments. The Editor therefore no longer has confidence in the integrity of the data in this article.

Author Guoying Zhang has stated on behalf of all co-authors that they agree to this retraction.

